# Genetic Variability of the Monkeypox Virus Clade IIb B.1

**DOI:** 10.3390/jcm11216388

**Published:** 2022-10-28

**Authors:** Fabio Scarpa, Daria Sanna, Ilenia Azzena, Piero Cossu, Chiara Locci, Silvia Angeletti, Antonello Maruotti, Giancarlo Ceccarelli, Marco Casu, Pier Luigi Fiori, Nicola Petrosillo, Massimo Ciccozzi

**Affiliations:** 1 Department of Biomedical Sciences, University of Sassari, 07100 Sassari, Italy; 2 Department of Veterinary Medicine, University of Sassari, 07100 Sassari, Italy; 3 Department of Medicine and Surgery, Unit of Clinical Laboratory Science, University Campus Bio-Medico of Rome, 00128 Rome, Italy; 4 Research Unit of Laboratory, University Hospital Campus Bio-Medico, 00128 Rome, Italy; 5 Department GEPLI, Libera Università Ss Maria Assunta, 00193 Rome, Italy; 6 Department of Public Health and Infectious Diseases, University Hospital Policlinico Umberto I, Sapienza University of Rome, 00161 Rome, Italy; 7 Infection Prevention and Control, University Hospital Campus Bio-Medico, 00128 Rome, Italy; 8 Unit of Medical Statistics and Molecular Epidemiology, University Campus Bio-Medico of Rome, 00100 Rome, Italy

**Keywords:** genetic diversity, monkeypox, lineage B.1, epidemiology, orthopoxviruses

## Abstract

Monkeypox is caused by a sylvatic, double-stranded DNA zoonotic virus. Since 1 January 2022, monkeypox cases have been reported to WHO from 106 Member States across six WHO regions, and as of 2 October 2022, a total of 68,900 confirmed cases, including 25 deaths, occurred. Here, by using a whole genome approach, we perform a genetic and phylodynamic survey of the monkeypox virus Clade IIb B.1, which is the lineage causing the current multi-country outbreak. Results suggest that outbreaks seem to be isolated and localized in several epidemic clusters with geographic consistency. Currently, monkeypox appears to be a virus with a flattened genetic variability in terms of evolutionary path, with a very slow rate of growth in the population size. This scenario confirms that the monkeypox virus lacks the evolutionary advantage, given by the high level of mutation rate, which is very strong in RNA viruses. Of course, constant genome-based monitoring must be performed over time in order to detect the change in its genetic composition, if any.

## 1. Introduction

Monkeypox is an infectious viral disease caused by the monkeypox virus [1], which affects humans with symptoms similar to smallpox but is less severe in nature [2]. Unfortunately, unlike smallpox, monkeypox has not been eradicated and remains endemic in sub-Saharan Africa [2]. The monkeypox virus is represented by two main clades: the former Congo Basin clade (also known as the Central African clade), now called Clade I, and the former West African clade, now called Clade II (https://www.who.int/news/item/12-08-2022-monkeypox--experts-give-virus-variants-new-names (accessed on 10 October 2022)). On 13 May 2022, WHO was notified of two confirmed cases and one not-confirmed case of monkeypox (from the same household) in the United Kingdom (https://www.who.int/emergencies/disease-outbreak-news/item/2022-DON383 (accessed on 10 October 2022)). As of 2 October 2022, 68,900 laboratory-confirmed cases of monkeypox have been reported to WHO from 106 countries in all WHO Regions (https://www.who.int/publications/m/item/multi-country-outbreak-of-monkeypox--external-situation-report--7---5-october-2022 (accessed on 10 October 2022)) (see Table 1 for details). This current multi-country outbreak is caused by the monkeypox virus belonging to Clade II, which is the less virulent and severe lineage [3]. This is also confirmed by the total number of deaths (25) from 1 January through 2 October 2022 (see Table 1). Indeed, Clade I presents a case-fatality ratio (CFR) higher than 10%, while Clade II’s CFR is lower than 1% [4]. However, although we are facing the less severe lineage, genomic surveillance is required in order to verify if this diffusion causes an enlargement of the genetic variability, the population size, for the number of lineages. The rapid diffusion that caused an outbreak is given by genomes belonging to the Clade IIb lineage B.1 [3].

In such a context, here, we performed a genetic and phylodynamic survey aimed at providing some key points on the genetic variability of the monkeypox virus Clade IIb lineage B.1 updated to all genomes publicly available on 10 October 2022. The research aimed to verify the occurrence of epidemic cluster in the Clade IIb B.1 (which is the most common lineage currently widespread) and the variation in terms of genetic variability from the first reported cases to current days. This kind of information is very important because it allows us to understand if the viral population size is growing or stationary during times.

## 2. Materials and Methods

The analyses were based on 1271 whole genomes downloaded from the GSAID database (available at https://gisaid.org/ (accessed on 2 October 2022)) (see Supplementary Materials). The update for the building of the dataset is 2 October 2022. Genomes were aligned by using the algorithm L-INS-I implemented in Mafft 7.471 [5], producing a dataset 199,972 bp long.

In order to avoid bias linked to the occurrence of deletions, insertions, and recombination events, the test Φ (or Pairwise Homoplasy Index, PHI) was applied, following Bruen et al. [6].

Population dynamics, based on genomes of monkeypox virus Clade IIb lineage B.1 (collection date range: 4 May 2022–14 September 2022), was reconstructed by implementing the evolutionary of 5 × 10^−5^ [4 × 10^−5^–6 × 10^−5^], proposed by Firth et al. [7] for variola viruses, by using the software Beast 1.10.4 [8] following Scarpa et al. [9].

In order to verify the temporal signal of molecular phylogenies, a tip-to-root regression was performed by using the software TempEst 1.5.3 [10]. The tree used for the analysis in TempEst was obtained by using the software IQ-Tree [11].

In addition, the genomic epidemiology of monkeypox virus Clade IIb lineage B.1 has been reconstructed by using the next strain available at https://gisaid.org/hmpxv-phylogeny/, last updated 2 October 2022.

## 3. Results and Discussion

The Φ statistic test suggested the lack of events attributable to deletions, insertions, and recombination. The Φ test, which is based on the principle of compatibility [6], indicated a condition of compatibility for all of the parsimony informative sites pairwise tested.

**Figure 1:**
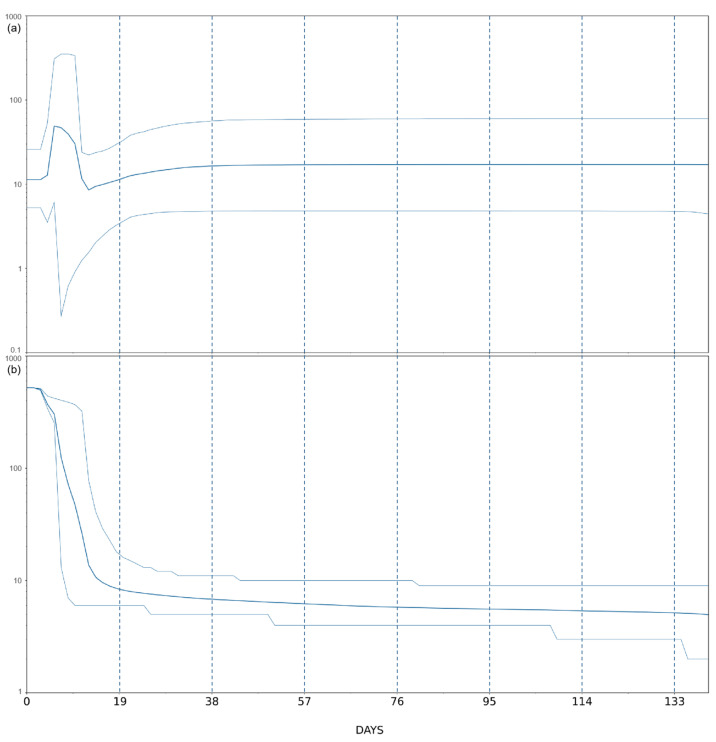
(**a**) Bayesian Skyline Plot of Monkeypox virus Clade IIb B.1. The viral effective population size (y-axis) is shown as a function of days (x-axis). (**b**) Monkeypox virus Clade IIb B.1 variant lineages through time. Number of lineages (y-axis) is shown as a function of days (x-axis). Thin lines represent the 95% high posterior density (HPD) region.

Population dynamic of monkeypox virus Clade IIb B.1 (Figure 1) suggests a general evolutionary path with flattened genetic variability. Indeed, Bayesian Skyline Plot (BSP) describes a condition where from the oldest isolate collected on 4 May 2022 to about late August, the genetic variability seems to have not undergone major increases, showing a stationary trend. At the end of August, a slight decrease occurred until the first days of September when the level of the genetic variability increased, reaching its peak around 4 September 2022 to then descend and stabilize on a new plateau after a few days. Accordingly, Figure 1b shows an increase in the number of lineages in correspondence with the reaching of the peak in early September. This trend is very different from such showed with SARS-CoV-2, which has significantly mutated over the course of the pandemic [12], producing many lineages and sub-lineages showing higher expansion capabilities [13].

**Figure 2:**
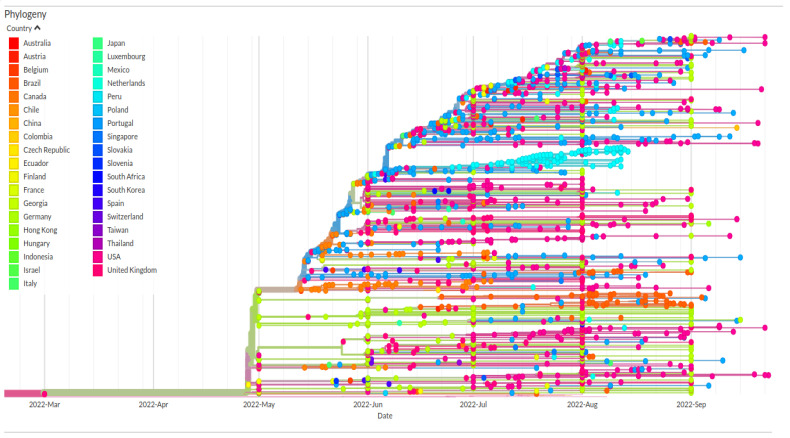
Highlight of the Monkeypox virus Clade IIb B.1 in the time-scaled phylogenetic tree of a representative global subsample of 1777 whole genomes from 37 countries (see Table 2) updated to 10 October 2022. Figure has been edited by using the software GIMP 2.8 (available at https://www.gimp.org/downloads/oldstable/ (accessed on 10 October 2022)).

This scenario is consistent with a very slow rate of growth in the population size. This point of view is also confirmed by the phylogenomic reconstruction (Figure 2), which indicates the occurrence of several small little clusters almost independent of each other. In a few cases, it seems that some specimens belonging to a given cluster go out of the cluster, connecting other clusters in a general epidemic-type cluster with a closed community, with a trend very similar to the seasonal flu one (e.g., see Mugosa et al. [14]). Indeed, the geographically based genetic structure allows one to associate a given cluster with its geographic area of origin (see Table 2 for details on countries of origin). In addition, localized clusters appear as evolutionary blind lineages with few or no descendants, and the branches’ length suggests low diversification.

This condition has also been confirmed by the test for the temporal signal, performed with a tip-to-root regression, which indicates a lack of a positive association between sequence divergence and sampling dates (Correlation Coefficient: −0.2246; R Squared: 5.0447 × 10^−2^). The causes of this result can be multiple [15], but in this case, the absence of a temporal signal is probably caused by the poor differentiation and very low genetic variability within the analyzed dataset, which is common in DNA viruses [7], which evolve far more slowly than RNA viruses, causing a weak or absent association with divergence and sampling date. The monkeypox virus lacks the evolutionary advantage, given by the high level of mutation rate, which is very strong in RNA viruses. In addition, it should be pointed out that in the analyzed dataset, which includes all genomes worldwide collected in about four months, the largest genetic distance found amounts to 0.005 (±0.0002). However, it is interesting to note that among DNA viruses, there are several exceptions (such as herpesviruses, for instance) that present levels of genomic variability very similar to many RNA viruses [16]. For instance, cytomegalovirus is known to present considerable inter-host and intra-host genetic divergence across tissue compartments and times of infection (see Delmotte et al. [17] and reference therein).

As far as human health is concerned, the biological condition of the monkeypox virus is a plus because it does not allow the virus to have a rapid spread, such as SARS-CoV-2 or many other RNA viruses [12]. Indeed, after several months, the monkeypox virus has still not exploded in terms of population size and contagiousness, and as highlighted in the WHO week report, during the week of 26 September to 2 October 2022, the number of monkeypox cases reported in the Regions of Europe and the Americas declined, driving the global downward trend observed since August 2022 (https://www.who.int/publications/m/item/multi-country-outbreak-of-monkeypox--external-situation-report--7---5-october-2022 (accessed on 10 October 2022)).

However, the monkeypox multi-country outbreak should not be underestimated, and molecular monitoring at the genome level must be constantly undertaken to acquire a more wholesome and well-rounded viewpoint. The importance of unstopped surveillance is also required because of the occurrence of asymptomatic infections, which may be unknown, and thus the total number of infections can be underestimated [18]. The virus may have emerged in early March 2022, and the distinct cluster in the phylogenomic reconstruction of Monkeypox suggests an early and cryptic spread of the virus [3,19], which now must be kept under control. Indeed, it should be highlighted that although slow, the evolutionary rate of lineage B.1 is faster than other lineages [19]. Moreover, in a recent study, it has been observed an accelerated evolution of human MPXV is potentially driven by the action of APOBEC3 [4].

In conclusion, the data herein suggest that the multi-country outbreak caused by the monkeypox virus seems to be localized in several epidemic clusters with geographic consistency. However, the current low capability in the differentiation of the monkeypox virus must not be understood as a reason to let down the guard against the outbreak. On the contrary, these findings further suggest the importance of a constant genome-based survey.

## Figures and Tables

**Table 1:** Number of cumulative confirmed monkeypox cases and deaths reported to WHO (https://www.who.int/publications/m/item/multi-country-outbreak-of-monkeypox--external-situation-report--7---5-october-2022 (accessed on 10 October 2022)) by WHO Region in 106 countries, from 1 January 2022 to 2 October 2022.

WHO Region	Confirmed Cases	Deaths
African Region	714	13
Region of the Americas	43,181	6
Eastern Mediterranean Region	64	1
European Region	24,737	4
South-East Asia Region	23	1
Western Pacific Region	181	0
World	68,900	25

**Table 2:** Country of origin of the 1777 genomes included in the phylogenetic analysis showed in Figure 2.

#	Countries	Number of Genomes	#	Countries	Number of Genomes
1	Australia	1	20	Japan	1
2	Austria	1	21	Luxembourg	2
3	Belgium	16	22	Mexico	2
4	Brazil	97	23	Netherlands	31
5	Canada	103	24	Peru	131
6	Chile	1	25	Poland	1
7	China	1	26	Portugal	327
8	Colombia	14	27	Singapore	10
9	Czech Republic	1	28	Slovakia	12
10	Ecuador	1	29	Slovenia	7
11	Finland	2	30	South Africa	2
12	France	22	31	South Korea	1
13	Georgia	1	32	Spain	9
14	Germany	305	33	Switzerland	6
15	Hong Kong	1	34	Taiwan	2
16	Hungary	2	35	Thailand	4
17	Indonesia	1	36	USA	471
18	Israel	1	37	United Kingdom	171
19	Italy	16			

## Data Availability

Genomes analyzed in the present study were taken from the GSAID database and are available at https://gisaid.org/ (accessed on 2 October 2022).

## Supplementary Materials

The following supporting information can be downloaded at: https://www.mdpi.com/2077-0383/11/21/6388/s1.
